# Dietary Intake Adequacy and Food Sources of Nutrients Involved in the Methionine-Methylation Cycle in Women of Childbearing Age from the ANIBES Spanish Population

**DOI:** 10.3390/nu13092958

**Published:** 2021-08-25

**Authors:** Marina Redruello-Requejo, Alejandra Carretero-Krug, Paula Rodríguez-Alonso, María Lourdes Samaniego-Vaesken, Teresa Partearroyo, Gregorio Varela-Moreiras

**Affiliations:** 1 Departamento de Ciencias Farmacéuticas y de la Salud, Facultad de Farmacia, Universidad San Pablo-CEU, CEU Universities, Urbanización Montepríncipe, Alcorcón, 28925 Madrid, Spain; m.redruello@usp.ceu.es (M.R.-R.); ale.carretero.ce@ceindo.ceu.es (A.C.-K.); l.samaniego@ceu.es (M.L.S.-V.); t.partearroyo@ceu.es (T.P.); 2 Grupo USP-CEU de Excelencia “Nutrición Para la Vida (Nutrition for Life)”, ref: E02/0720, Alcorcón, 28925 Madrid, Spain; 3 Fundación Española de la Nutrición, 28010 Madrid, Spain; prodriguez@fen.org.es

**Keywords:** choline, betaine, vitamin *B_6_*, folates, vitamin *B_12_*, micronutrients, dietary survey, food sources, fertile, Spain

## Abstract

Growing evidence confirms choline as a critical perinatal nutrient. However, intake levels of choline and betaine among the Spanish fertile population remain unknown. Given their role in one-carbon metabolism with potential epigenetic effects, the aim of the present study was to evaluate the dietary intakes, their adequacy to existing guidelines and the main food sources together with other micronutrients involved in the methylation-methionine cycle (vitamin B_6_, folates and vitamin B_12_) in women of childbearing age. The ANIBES study, a cross-sectional study of a representative sample of women of childbearing age (18–45 years, *n* = 641) resident in Spain, was used. The sample was divided into younger women (18–30 years, *n* = 251) and older women (31–45 years, *n* = 390). Dietary intake was assessed by a three-day dietary record by using a tablet device. Total median intakes for the total sample were 303.9 mg/d for choline; 122.6 mg/d for betaine; 1.3 mg/d for vitamin B_6_; 140.8 μg/d for folates, and 3.8 μg/d for vitamin B_12_. The older subgroup showed significantly higher choline (*p* < 0.05), betaine (*p* < 0.001) and folates (*p* < 0.05) intakes than younger women. Main food sources for the whole sample were meat and meat products for choline (28.3%), vitamin B_6_ (25.7%) and vitamin B_12_ (22.8%); cereals and derivatives (79.9%) for betaine; vegetables (20.0%) for folates. Overall intake adequacy was only observed for vitamin B_12_, with a very limited number of participants showing adequate intakes for all the other micronutrients. These results illustrate there is a relevant need to raise awareness about optimizing the status of the methionine cycle-related vitamins and cofactors in this potentially vulnerable population.

## 1. Introduction

The importance of ensuring an adequate status during pregnancy from the nutrients (choline, betaine, vitamin B_6_, folates and vitamin B_12_) involved in one-carbon metabolism via the methionine-methylation cycle is well established. However, while dietary intake recommendations, and even supplementation protocols, are well defined for B vitamins [1,2,3], this is not the case for choline and betaine.

Previous results have indeed shown that prevalence rates of infants born with neural tube defects (NTDs) have successfully decreased since the implementation of supplementation and fortification protocols with synthetic folic acid (FA) [4]. However, NTDs continue to occur and available data suggests that 5–6 cases per 10,000 pregnancies represent the lowest prevalence that is achievable through these current protocols, indicating that a percentage of the remaining NTDs are not sensitive to FA [4,5]. Recent intervention trials showed that about 70% of common and severe NTDs are preventable by periconceptional (at least one month before conception and at least two months after conception) supplementation with FA, a percentage that rose to 90% when additional supplementation with the vitamins riboflavin (vitamin B_2_), pyridoxine (vitamin B_6_) and cobalamin (vitamin B_12_) was incorporated [6]. One explanation for this phenomenon may be the elevated plasma total homocysteine (pHcy) levels observed in mothers of children with NTDs [7,8,9]. Data derived from the UK National Diet and Nutrition Surveys (NDNS 2000/2001 and 2008/2012) show the high prevalence of hyperhomocysteinemia (≥12 μmol/L) in British women of childbearing age (19–39 years), amounting to 21.2% [10]. High pHcy is also traditionally linked to a wide variety of negative health outcomes such as ischemic heart disease, cerebrovascular events, cognitive impairment or dementia [11]. It is known that there is a direct relationship between pHcy and plasmatic levels of FA, but also with other compounds such as vitamins B_6_ and B_12_, choline and betaine.

FA supplementation is now widely used for the control of pHcy levels [12], for the prevention and treatment of macrocytic or megaloblastic anemia [13], and as previously mentioned, in pregnant women for the prevention of congenital malformations, particularly NTDs [14], in what is perhaps one of the greatest example of generalized supplementation implemented by public health policies. During pregnancy there is an increase in cell division, which is associated with an acceleration of carbon transfer reactions, including nucleotide synthesis and cell division, leading to an increase in FA requirements [14] and making folates a key nutrient to control during this physiological situation. However, the available evidence indicates intake of folates is insufficient for the vast majority of the Spanish population. Vitamins B_6_ and B_12_ share some key functions related to one-carbon metabolism, most notably for their direct role in regulating pHcy levels by acting as coenzymes in their metabolization to cysteine and methionine, respectively [12]. Vitamin B_6_ deficiency has been associated with microcytic anemia, seborrheic dermatitis, epileptiform seizures, depression and confusion [15]. Vitamin B_12_ deficiency is traditionally associated with megaloblastic or macrocytic anemia and neurological disorders [16], but in addition recent evidence indicates that low maternal vitamin B_12_ is a significant predictor, independent of FA, of NTDs risk [17]. In Spain, the recommended intakes defined during pregnancy are slightly increased with respect to those of non-pregnant women, being 1.9 mg/day for vitamin B_6_ and 2.2 µg/day for B_12_ (respectively, 0.3 mg/day and 0.2 µg/day more than those indicated for non-pregnant women) [1].

Because both choline and FA show a metabolic crossover, it has been hypothesized that insufficient choline intakes could be also associated with NTDs [18]. So far, the nutritional and health benefits of FA could have been eclipsing those of choline, which has been largely ignored in prenatal nutrition [19], but pre- and postnatal choline availability has indeed been identified to be relevant for neurodevelopment in animal studies [20]. In fact, choline is an essential nutrient that is also a precursor of four key biological compounds. It is a precursor of phosphatidylcholine, which accounts for about 50% of phospholipids in mammalian membranes [21] and which is also involved in very low-density lipoprotein (VLDL) assembly in the liver [22]. In addition, it is from phosphatidylcholine that sphingomyelin, a component of the myelin sheath that covers neuronal axons, is formed. Furthermore, choline is acetylated in cholinergic neurons to form the neurotransmitter acetylcholine [23]. Finally, in the methylation-methionine cycle, betaine is the metabolite through which choline is involved in the folate-dependent one-carbon metabolism. Specifically, betaine acts as a methyl-group donor for the regulation of pHcy through its remethylation into methionine. The result of these reactions is the production of S-Adenosylmethionine (SAM), the universal methyl-group donor and a key agent in the regulation of gene expression or epigenetics [24]. Additionally, betaine is also an osmolyte, which helps to maintain cell volume and to prevent protein unfolding [23].

As demonstrated, choline and betaine play key physiological processes in cell division, nervous system development and epigenetic regulation, being particularly necessary for fetal brain development and placental function [25,26,27,28]. These findings make them crucial nutrients to monitor during pregnancy, or better yet, to optimize in the nutritional status of women intending to become pregnant. Studies have already shown that choline supplementation reduces the risk of preeclampsia [29], preterm birth [30] and other non-neural birth defects such as orofacial clefts, urethral and diaphragmatic malformations [31,32,33]. As for NTDs, several observational studies show an association between adequate maternal choline intake and reduced risk of NTDs [25,26,27,28]. The explanation for these observations is that the metabolic crossover in the folate and methionine-methylation cycle represents a compensatory mechanism to maintain sources of one-carbon units during folate or choline deficiency [23]: during transient periods of FA deficiency, choline, as a betaine precursor, may compensate by allowing homocysteine remethylation, thereby increasing choline and betaine dietary requirements. Conversely, during choline deprivation leading to low betaine content, homocysteine remethylation is maintained from 5-methyltetrahydrofolate, increasing folate requirements as well. While the first assumption should be avoided with current FA supplementation protocols, the assumption of a choline deficiency that could be masked by this same FA supplementation practices cannot be ruled out. Nor can its consequences: in the face of persistently insufficient choline levels, FA will be employed in the methionine-methylation cycle to support one-carbon metabolism, diverting it from its critical role in DNA synthesis and repair [19]. Interestingly, the fact that 5-methyltetrahydrofolate and choline/betaine may be considered as partially interchangeable methyl group carriers was described as early as 1992 [34]. Of importance, protocols based on FA supplementation for the prevention of NTDs seem to be adequate, but their effectiveness may be reduced if sufficient choline and betaine intakes are not ensured as well.

Due to the scarce availability of data on their intake and health consequences, both choline and betaine are not suitable for the setting of average requirements and recommended dietary intakes (RDI). Only in the case of choline has the Panel on Dietetic Products, Nutrition and Allergies (NDA) of the European Food Safety Authority (EFSA) [35] established reference values, namely in the form of adequate intakes (AIs) or estimates of the level of intake that appears sufficient for virtually the entire population. For all adults, the panel established an AI of 400 mg/day. For pregnant women, the Panel derived an AI of 480 mg/day, calculated by extrapolation from the AI for non-pregnant women and the mean gestational increase in body weight. For lactating women AI was set at 520 mg/day, as the amount of choline secreted per day in human milk during the first six months of exclusive breastfeeding (120 mg/day) is added to the AI for non-lactating women.

The fact that these AIs for choline are even higher for the lactation period than during pregnancy adds to the reasons for potentially considering a supplementation protocol for this micronutrient in pregnant women, if not in fertile women, similar to the ones applied for FA. In this regard, the American Medical Association (AMA) and the American Academy of Pediatrics (AAP) both advocate the addition of choline to prenatal pills and infant formula [36,37]. In Europe, however, equivalent bodies still do not provide indications for maternal choline supplementation because of the limited risk/benefit assessment studies that usually lead Europe to follow the precautionary principle. In addition, the panel discusses the importance of considering the effects of genotypes when establishing intake recommendations and possible supplementation protocols, as several single nucleotide polymorphisms (SNPs) in genes encoding enzymes involved in choline and methyl-group metabolism—some of which are present at a frequency of up to 70% in mixed populations—may alter choline requirements [35]. In this regard, it has been suggested that SNPs in genes that regulate choline metabolism could be possible risk factors for NTDs [38].

Nonetheless, the first step towards addressing these issues requires knowledge on the current intake situation of the population, and while those of the B vitamins have been assessed for some time as already mentioned, this is not the case for choline and betaine, whose intakes remain unknown in Spain. Other studies carried out in Western populations have identified meat, milk, grain, egg and their derived as the main food sources of choline [39] and cereal foods for betaine [40].

This is the first attempt to assess choline and betaine intakes amongst the Spanish population and given their involvement in the one-carbon cycle with possible epigenetic effects, that evaluation in women of childbearing age may be of particular relevance. Considering their interrelationship, the intake of the other B vitamins involved in one-carbon metabolism has also been evaluated.

For all the aforementioned, the aim of the present study was to evaluate the dietary intakes, their adequacy to existing guidelines and the main food sources of the micronutrients involved in the methylation-methionine cycle (choline, betaine, vitamin B_6_, folates and vitamin B_12_) in a representative sample of women of childbearing age (18–45 years) resident in Spain.

## 2. Materials and Methods

The design, protocol, and methodology of the ANIBES study have been already described in detail elsewhere [41,42].

The determination of the average intake levels of each micronutrient was carried out using the food consumption data available from the ANIBES study, but not on the consumption of dietary supplements [41], a cross-sectional study conducted over 3 months (September to November 2013) in a representative sample from the Spanish population (9–75 years, *n* = 2009). For the present study, we specifically used the data from women of childbearing age (18–45 years, *n* = 641) divided into two groups: younger women (women of childbearing age (18–30 years, *n* = 251)) and older women (women childbearing age (31–45 years, *n* = 390)). Participants recorded all foods and drinks consumed over 3 days by means of a tablet device which allowed for comprehensive information as well as photograph collection of all consumed meals (at home and outside). To represent all days of the week equally, participants recorded their intakes during two weekdays and one weekend day. Food records were returned from the field in real time, to be coded by trained personal and supervised by dieticians. Data obtained from food manufacturers and nutritional information provided on food labels were also included. A photographic food atlas was used to assist in assigning gram weights to portion sizes. Food, beverage, energy, and nutrient intakes were calculated from food consumption records using the VD-FEN 2.1 software, a dietary evaluation program from the Spanish Nutrition Foundation (FEN), Spain, which was updated for the ANIBES study based mainly on the Spanish Food Composition Tables [1]. Sample quotas were established regarding level of education, habitat size and geographical distribution according to Nielsen areas (Table 1).

### 2.1. Calculation of Nutrients Consumption and Adequacy of Intakes

From the population mean of consumption of each food in grams/day, the contribution of the nutrients was estimated by calculating values individually for each food according to its nutritional composition. To determine the nutritional composition of those foods or recipes that are most typical of the eating habits of the Spanish population, an estimate was made from the ingredients comprising them.

To calculate the contribution of folates and vitamins B_6_ and B_12_, the nutritional composition data collected in the Spanish Food Composition Tables were used [1]. In the case of foods with some micronutrient not included in those tables, it was substituted by a determined value in a food of similar nutritional composition, or an average was assumed at the level of the group and/or subgroup of foods.

Choline and betaine are currently not included in the national food composition databases in Europe. Therefore, it was necessary to refer to the National Nutrients Database for Standard Reference from 2018, published by the USDA [43]. For choline, the data for total choline was used, as the sum of free choline, glycerophosphocholine, phosphocholine, phosphatidylcholine, and sphingomyelin. In the cases of foods with any micronutrient not determined in these tables, the database of choline and betaine content for the most common foods was first consulted, also elaborated by the USDA but pertaining to the year 2008 [44]. Faced with the same absence of determinations, missing values were substituted for a determined value in a food with a similar nutritional composition or an average was assumed at the group and/or subgroup level of foods.

Available RDIs and AIs for Spain, Europe and USA were used to compare the actual reported intake with those recommended (Table 2). Intake adequacy of choline, vitamin B_6_, folates and vitamin B_12_ were expressed as the percentage of population achieving >80% of the RDI (% above 80% RDI) for each micronutrient according to Moreiras et al. [1], EFSA [2] and IOM [3].

The final protocol of the ANIBES Study was approved by the Ethical Committee for Clinical Research of the Region of Madrid, Spain. The study was coded as “FEN 2013” and approved on 31 May 2013 [45].

### 2.2. Statistical Analysis

Results are expressed as median (interquartile range) or as percentage. To establish if the samples were parametric or non-parametric, the Kolmogorov–Smirnov test was used. The non-parametric data were statistically analysed by Mann–Whitney’s U test. The values obtained for the three RDI (Spanish [1], EFSA [2] and IOM [3]) were compared using chi-square test. Differences were considered significant at *p* < 0.05. Data analysis was performed with SPSS 27.0 software package (IBM Corp., Armonk, NY, USA).

## 3. Results

A sample of 641 women of childbearing age participated in the study, with ages ranging from 18 to 45 years (40.1% between 18–30 years, younger women, and 59.9% between 31–45 years, older women). Median daily choline intakes for Spanish women of childbearing age (18–45 years) are shown in Table 3. Older women had significantly higher choline total intakes (311.8 mg/day) than younger women (292.4 mg/day) (*p* < 0.05). In addition, prevalence of adequacy for choline intakes (% population above 80% RDI) in the study population is presented by age-group in Table 3 according to the different international RDI: EFSA [2] and IOM [3]. The prevalence of adequacy for choline in the total study population was 39.5%, and 35.1% according to the EFSA, and IOM RDI criteria, respectively (Table 3). The proportion of adequacy for total choline intakes in younger women was 35.1% and 31.1%, and for older women it was 42.3% and 37.7% according to the EFSA and IOM references, respectively (Table 3).

Betaine intakes are shown in Table 4. Older women had significantly higher betaine intakes than younger women (*p* < 0.001). Total median betaine intakes amongst younger women were 113.5 mg/day while for older women was 130.2 mg/day.

Table 5 shows vitamin B_6_ intakes in women of childbearing age from the ANIBES study. Median vitamin B_6_ intakes were 1.3 (1.0–1.7) mg/day across the whole sample (Table 4). No significant differences were observed regarding vitamin B_6_ intakes within age-groups. Based on the different RDI criteria, prevalence of adequacy for vitamin B_6_ in the total study population was 52.1%, 59.3%, and 79.4% according to the Spanish, EFSA, and IOM RDI criteria, respectively (Table 4). For total women of childbearing age and both age-groups, IOM RDI criteria estimated a significantly higher prevalence of adequacy for vitamin B_6_ when compared to the Spanish and EFSA criteria (*p* *≤* *0.05*). In addition, the prevalence of adequacy for vitamin B_6_ estimated by the EFSA criteria was significantly higher than that estimated by the Spanish criteria in total and older women(*p* *≤* *0.05*) (Table 5).

Table 6 displays the reported daily intake levels of folates, which increased with age. Higher reported intake was observed in older women (148.3 µg/d) compared with younger women (131.4 µg/d) (*p* < 0.05). Moreover, potential prevalence of adequacy for folates (% population above 80% RDI) in the total study population according to the national and international RDI is also presented by total population and age-group in Table 6. The proportion of adequacy for folates in total women population was 2.3%, 21.4% and 2.3% according to the Spanish, EFSA and IOM RDI criteria, respectively. Data analysis stratified by age-group revealed that the prevalence of adequacy for folates based on the Spanish and IOM RDI were significantly lower than the prevalence results obtained using the EFSA RDI, in all cases.

Median vitamin B_12_ intakes across the total population of women of childbearing age were 3.8 (2.5–5.3) µg/day, while for younger women it was 3.7 μg/day and for older women was 4.0 μg/day (Table 7). Vitamin B_12_ adequacy in total women of childbearing age was 91.0% 62.2% and 86.1% according to the Spanish, EFSA and IOM RDI references, respectively. Vitamin B_12_ adequacy in the total population was significantly higher according to the Spanish than IOM and EFSA RDI (*p* < 0.01) and in IOM vs. EFSA RDI (*p* < 0.01). However, vitamin B_12_ adequacy in age-group was significantly higher according to the Spanish and IOM than EFSA RDI (*p <* 0.01).

### Contribution of Food and Beverage Groups to Choline, Betaine, Vitamin B_6_, Folates and Vitamin B_12_ Intakes

**Figure 1:**
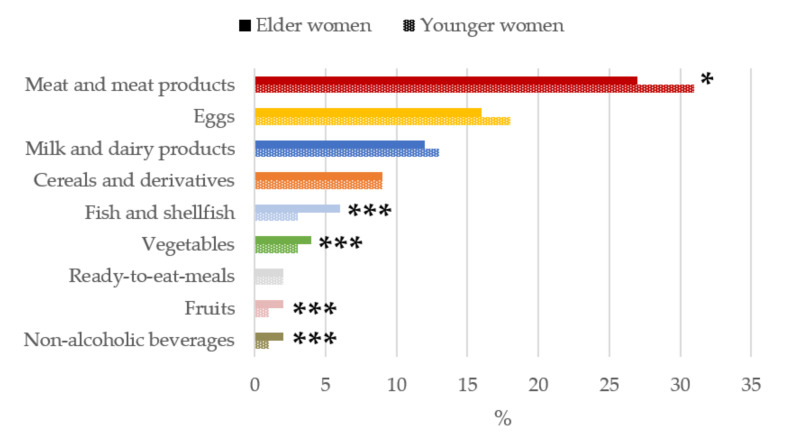
Contribution of main sources (food and beverages) to choline intake in women of childbearing age from the ANIBES study by age-group. * *p* < 0.05 difference vs. younger women (Mann–Whitney’s U test). *** *p* < 0.001 difference vs. younger women (Mann–Whitney’s U test).

**Figure 2:**
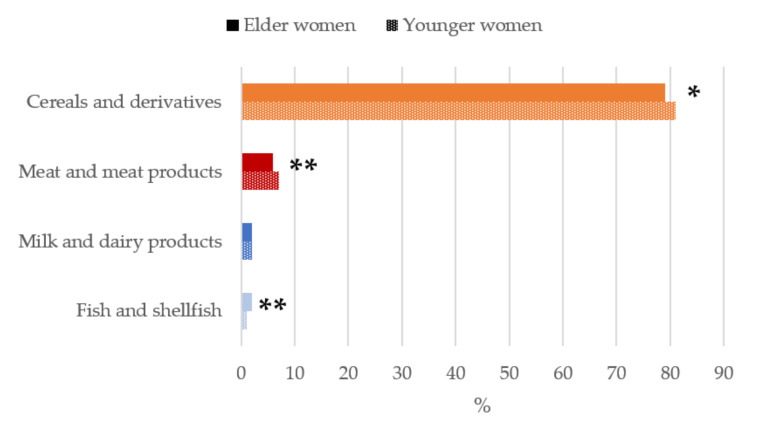
Contribution of main sources (food and beverages) to betaine intake in women of childbearing age from the ANIBES study by age-group. * *p* < 0.05 difference vs. younger women (Mann–Whitney’s U test). ** *p* < 0.01 difference vs. younger women (Mann–Whitney’s U test).

**Figure 3:**
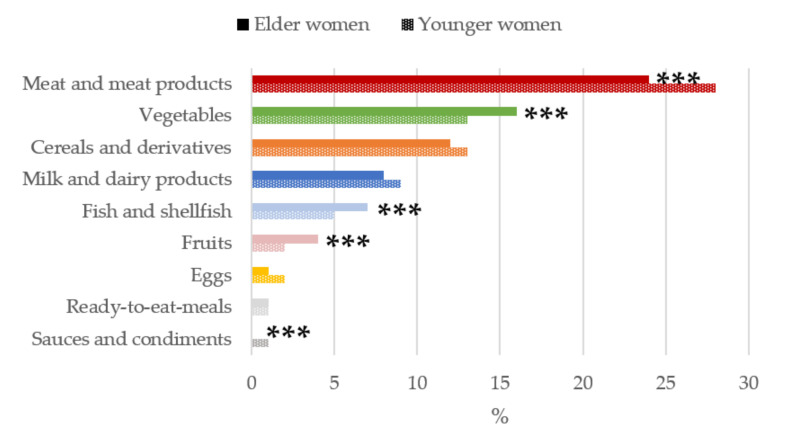
Contribution of main sources (food and beverages) to vitamin B_6_ intake by age-group in women of childbearing age from the ANIBES study. *** *p* < 0.001 difference vs. younger women (Mann–Whitney’s U test).

**Figure 4:**
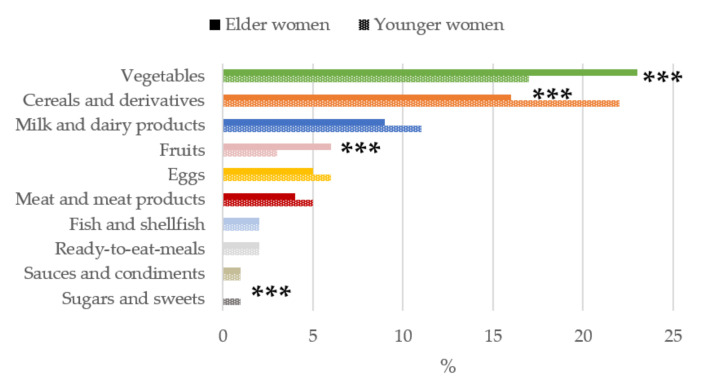
Contribution of main sources (food and beverages) to folates intake by age-group in women of childbearing age from the ANIBES study. *** *p* < 0.001 difference vs. younger women (Mann–Whitney’s U test).

**Figure 5:**
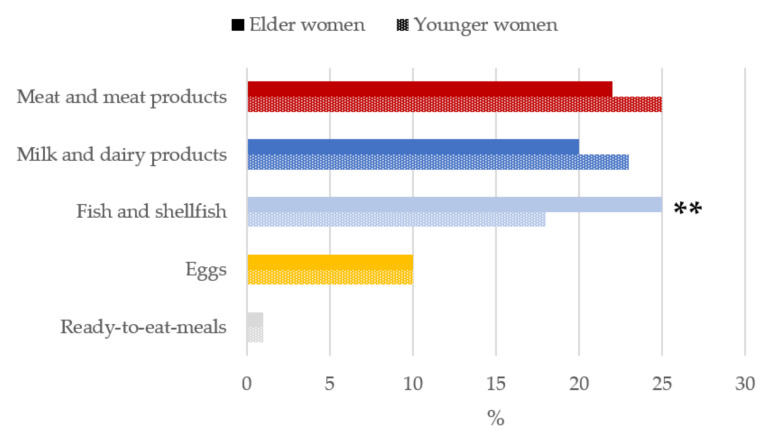
Contribution of main sources (food and beverages) to vitamin B_12_ intake by age-group in women of childbearing age from the ANIBES study. ** *p* < 0.01 difference vs. younger women (Mann–Whitney’s U test).

It is worth highlighting that, as shown in Figure 1 Figure 2 Figure 3 Figure 4 Figure 5, only those foods which contributed at least 1% to nutrient intakes of the population have been included. The contribution (%) of food and beverage categories to the daily choline intake is shown, categorized by age-group, in Figure 1. Meat and meat products were the main contributors of choline for the entire sample, where they were significantly higher in younger than older women (*p* < 0.05). Eggs were the second largest contributors followed by milk and dairy products.

When analyzing the contribution of food and beverage main food group sources to daily betaine intakes (Figure 2), the highest median proportional contribution in both older and younger women were firstly cereal and derivatives (79%–81%), being significantly higher (*p* < 0.05) in the younger group followed by meat and meat products (6%–7%), of which intakes were significantly higher in youngers (*p* < 0.01). Thirdly, milk and dairy products accounted for a 2% of betaine intakes. Together, these three food groups contributed to >85% of betaine intakes of the studied population.

The main sources of vitamin B_6_ for women of childbearing (Figure 3) were meat and meat products, with a significantly higher contribution for the younger women group (28%) compared to older women (24%). Older women consumed a higher proportions of vegetables than younger women (16% vs. 13%). Cereals and derivatives ranked third (12%), and next were milk and dairy products (8%). All of these above groups contributed to nearly 60% of the vitamin B_6_ reported intake.

The contribution of food and beverage groups to the daily folates intakes is shown in Figure 4. The food groups with the highest median proportional contribution to total folates intakes in both young and older women were vegetables and cereals and derivatives. In contrast, when participants were compared according to age group, we found that in older women, vegetables and fruits were the main sources of folates, being significantly higher than the younger women group (*p* < 0.001). However, for younger women, the contribution of cereals and derivatives and sugar and sweets were significantly higher than in the older women group (*p* < 0.001).

Meat and meat products (25%), milk and dairy products (23%) and fish and shellfish (18%) were the main sources of vitamin B_12_ for younger women while in the case of the older women, fish, and shellfish (25%) were the greatest contributors, followed by meat and meat products (22%) and milk and dairy products (20%) (Figure 5).

## 4. Discussion

The present assessment of nationally representative data encompasses a novel insight into betaine and choline intakes of women of fertile age in the Spanish population. In addition, the adequacy of intakes of vitamins B_6_, folates and B_12_ were studied specifically for the fertile women age segment. The major dietary contributors of betaine, choline and the cited vitamins among these group’s diet were also identified.

### 4.1. Dietary Intake Adequacy

Overall, average intakes for all nutrients were well below recommendations, except for vitamin B_12_ [1,2,3,35]. Although discouraging, the results obtained are consistent with the available evidence. At the European level, Venneman et al. [39] assessed choline intakes from populations across 12 representative surveys from 9 different countries. Average intake estimates ranged from 291 to 374 mg/d among females aged 18 to ≤65 years old, values that were well below the AI for choline. Worryingly, Mediterranean countries showed the lowest intakes. Wallace et al. [46] also found suboptimal intakes of choline to be prevalent across the United States, with only 6.1% of female participants in the 2009–2012 National Health and Nutrition Examination Survey (NHANES) achieving the AIs. In the present study intakes observed for the Spanish female population of childbearing age are within the range observed by Vennemann et al. [39] in adult European women and slightly higher than those of female participants in the NHANES study in the USA, where, as in the present study, younger adult women (19–30 years) showed lower intakes than older women (31–50 years), respectively 250 mg/d and 278 mg/d [46]. Anyhow, the adequacy levels for the Western population are suboptimal. In the present study, less than half of the population showed sufficient choline intake levels, i.e., above 80% of the AI. However, the choline intake must be evaluated with caution, as intake levels above the AI imply a low probability of inadequate intake, but intakes below the AI does not necessarily indicate inadequacy [47]. Still, the small number of women achieving average intakes close to the AI is a matter of concern.

As for betaine, the uncertainty about intake levels in the Spanish and European population is even greater. In fact, the only study evaluating betaine intake in the European population is that conducted in 2010 by Prince et al. [48] in 79 adults (45–65 years) of Northern Ireland, with mean betaine intake at 127 mg/day. Outside Europe, Ross et al. [40] reviewed 17 studies from 9 different countries and adjusted data on betaine content in foods when the 2004 United States Department of Agriculture (USDA) database was used, reporting a mean betaine intake of 119 mg/day for women. The mean betaine intake observed in the present study is in line with the studies discussed above. However, the prevalence of adequacy cannot be evaluated as there are no established recommendations at present.

Concerning dietary intakes of B vitamins, there is an acceptable degree of evidence available at Spanish and European level. Compared with global results for all female participants (9–75 years; *n* = 996) included in the ANIBES Study, which showed mean intakes of 1.45 mg/day for vitamin B_6_, 156.3 µg/day for folate and 4.0 µg/day for vitamin B_12_ [49,50], our results for the subset of women of childbearing age are slightly lower (0.15 mg/day; 15.5 µg/day and 0.2 µg/day less, respectively). Lower intakes are also observed when comparing with data from Spanish adolescent females (14–17 years; *n* = 118) from the ENALIA (“National Dietary Survey on the Child and Adolescent Population”) Study (0.5 mg/day; 101.9 µg/day and 0.7 µg/day less, respectively) [51] and European adolescent females (15- < 19 years; *n* = 357) from the HELENA (“Healthy Lifestyle in Europe by Nutrition in Adolescence”) Study (0.2 mg/day; 54.2 µg/day and 0.9 µg/day less, respectively) [52], both obtained from two non-consecutive 24 h dietary recalls. Notably, in the case of folates, intakes observed in the present study were significantly lower in younger women than older women. Another cross-sectional study conducted by Planells et al. [53] in southern Spain and based on a 48 h dietary recall, also found that mean folates intakes were lower (by 10.2 µg/day) in younger women (25–39 years) compared to older women (40–49 years). When studying the level of adherence to the recommendations for B vitamins, the percentages of adequacy observed for this sample of women of childbearing age are also somewhat lower than in the other studies mentioned. Compared to the whole sample of the female population included in the ANIBES study, in the case of vitamin B_6_ the population reaching adequate intakes is reduced by approximately 25%. Similar observations are obtained when comparing with the adequacy reported for the adolescent population in the ENALIA and HELENA studies. In the case of vitamin B_12_, this reduction in the prevalence of adequacy is much less marked [49,50,51,52]. However, the different methodology used in studies evaluating dietary intakes makes a comparison between studies difficult where discrepancies, and even similarities, may be casual.

The situation of non-compliance with recommendations for folates is particularly worrying, as less than 3% of the population studied complied with the Spanish and US guidelines. These results are consistent with the adequacy level observed for the general female population included in the ANIBES study, which also remained at a low 3% [49]. It is worth mentioning that folates intake from these ANIBES studies were assessed only with food composition data from natural food sources. Voluntarily fortified products were not identified and therefore folic acid intakes of the population could not be derived from our results. In addition, the consumption of nutritional supplements was not assessed. Results from Spanish female adolescents included in the ENALIA study showed higher mean intake for folates, but still prevalence of adequacy remained at 8.7% of the sample [51]. In any case, these observations underline the need to optimize the nutritional status of the Spanish population and of this vulnerable group in particular, which should achieve an optimal nutritional status in folates. In pursuit of this goal, some authors affirm that efforts to increase folates intake through natural folate-rich foods are unlikely to be effective for health prevention on a population basis and would leave a large portion of the population, particularly women of reproductive age, at increased risk for diseases related to the vitamin insufficiency [54]. Moreover, Tabacchi et al. [55] in a review concerning the adequacy of micronutrients intake in Europe, observed that folates inadequacy across eight countries encompassed about 25% of the adult female population, according to the particular dietary reference intakes considered by the investigators. However, the level of inadequacy rose to almost 75% of these women when using the folates estimated average requirement cut-off value of 320 μg/d.

### 4.2. Main Food Sources

Detailed information on food sources is essential to better understand the strengths/weaknesses and quality of the diet of women of childbearing age in the Spanish population. Specifically, our results show that the highest percentages of choline are provided by meat and meat products, with a total contribution of around 28% of the estimated overall consumption of the sample. Similar findings were observed by Venneman et al. [39] in other European countries. It is important to note that the updated Dietary Guidelines recommend decreasing the intake of meat and derivatives as the Spanish diet contains an excessive amount of animal protein [56]. The present results show that animal food sources are still the main contributors to choline—with young women showing a significantly higher contribution from the meat and derivatives group—which is still not meeting the recommended AI. These findings highlight the urgent need to implement strategies to improve the intake and nutritional status of this component of great nutritional interest for the health of women of childbearing age and, consequently, their offspring. In this regard, it has already been noted that both the AMA and the AAP advocate the addition of choline to prenatal pills and infant formula [36,37]. There is growing evidence that choline supplementation might beneficially influence several physiological processes in the offspring (e.g., brain development, hypothalamic–pituitary–adrenal (HPA) axis stress reactivity) and improve overall offspring health (e.g., cognitive function, reduced risk of chronic and developmental diseases) [57]. Of interest, some authors suggest that fortification of staple products that are widely available and cost-efficient (e.g., grain-based or dairy products) and education on foods rich in choline may serve as an optimal strategy for increasing dietary intakes, without ruling out the need for nutritional supplementation for specific population groups such as pregnant and/or lactating women, seniors and vegans, as already noted in the case of vitamin B_12_ [46].

With regard to the food sources of betaine, the almost exclusive contribution observed for the cereals and derivatives group is in line with those described in the systematic review by Ross et at [40], where cereal foods provide approximately 60–67% of betaine in Western diets, vs. 20–40% in South-East Asian diets. These results emphasize the importance of wheat, specifically whole grain, as a source of betaine in the Western diet, and warn that those with celiac disease or gluten intolerance are likely to have low betaine intakes. However, pseudocereals such as quinoa, amaranth and buckwheat are also rich in betaine and/or choline and represent a good alternative dietary source of these compounds [40]. In addition, due to their high consumption levels, foods relatively low in betaine, such as beer, iced tea and ground beef, have become major betaine contributors among the US population [58].

The main sources of vitamin B_6_ for women of childbearing age from our study were meat and meat products, followed by vegetables and by cereals and derivatives. These results are similar to those observed for the Spanish population in the ANIBES study [50]. However, some differences are observed in comparison with the study carried out in Southern Spain by Planells et al. [53], where main B_6_ sources across their sample were firstly meat, with a similar contribution (27.6%), remarkably followed by fish (12.3%) and fruit (9.9%). In contrast, vegetables and cereals and its derivatives only accounted for 8.4% of vitamin B_6_ intakes each. The reason for these differences may be the fact that this study was published in 2002 and dietary habits have somewhat changed, as well as the fact that only the population of Southern Spain aged between 25 and 60 years old was included.

Regarding folates, the food groups with the highest proportional contribution to total intake were vegetables and cereals and derivatives. Again, these results are similar to those observed for the global ANIBES study population [49]. In contrast, Planells et al. [53] reported that fruits (25.8%), pulses (23.4%) and vegetables (20.4%) were the main folates sources, while cereals and derivatives only accounted for 3.6% (vs. 20.0% in the present study). Pulses are indeed a rich folates source, but according to our results this food group is amongst the lowest folates contributors to the Spanish diet. In this regard, other publications from the ANIBES study [45] and by Varela-Moreiras et al. [59] have confirmed the declining intakes of this staple food from the traditional Mediterranean diet.

Vitamin B_12_ is almost exclusively found in animal-derived foods and this is reflected in our results, as well in the global ANIBES study [49] which, however, differ from those obtained by Planells et al. [53], where major contributors to B_12_ intakes were meat accounting for 55.8% (vs. 22.8% in the present study), fish and shellfish with 23.8% (vs. 20.5%) and milk and dairy products with a reduced 6.8% (vs. 21.4%). Our data are in accordance with those obtained in other European studies in which the proportion of vitamin B_12_ obtained from meat and dairy products was higher than in the USA [60], where cereals are a notable contributor to vitamin B_12_ intake [61,62].

Finally, it should be noted that the significant differences observed by age group in the contribution of the main food sources to the intake of some of the micronutrients studied leads us to presume the existence of two different dietary patterns for each age group. On the one hand, younger women recorded a significantly higher contribution to their micronutrient intake from meat and meat products, cereals and derivatives, sugars and sweets, and sauces and condiments. On the other hand, older women showed significantly higher contributions of fish and shellfish, vegetables, and fruits. These findings indicate that the dietary pattern observed for young women is further away from the traditional Mediterranean diet pattern, observations that have long been reported for the general Spanish population [63]. Furthermore, this finding may explain the fact that the reported daily intake levels of choline, betaine and folates increase with age, being significantly lower for younger women.

### 4.3. Strengths and Limitations

Amongst the strengths of this study, we can highlight the careful design, protocol, and methodology; the use of a representative sample of the Spanish population; the innovative methodology for compiling dietary data and dietary assessment method; as well as the extended and updated food database created that allowed to obtain data not only from major food groups but also subgroups of key interest. Conversely, a limitation is its cross-sectional design, which provides evidence for associations but not for causal relationships. In addition, a major disadvantage was the lack of composition data on choline and betaine from European foods. The choline and betaine composition data from the USDA database used in this assessment may not precisely reflect their content in foods as acquired and as consumed in Europe.

## 5. Conclusions

This is the first study to jointly estimate the choline, betaine, folates, vitamin B_6_ and vitamin B_12_ intakes amongst Spanish women of fertile age. Overall intake adequacy was only observed for vitamin B_12_, with a limited number of participants showing adequate intakes for all the other components. In addition, mean intakes of choline, betaine and folates were significantly lower for younger women. The Spanish female population, particularly younger women, would largely benefit from a dietary pattern more in line with the traditional Mediterranean diet with increased ingestion of green leafy vegetables, pulses, and seeds. Results illustrate the relevant need there is to raise awareness about optimizing the nutritional status of the methionine cycle-related vitamins in this vulnerable population, but also the need to prioritise and discuss nutritional supplementation policies for all these components, following the model carried out with folic acid, and considering benefit and risk. Further work to improve and expand current food composition databases is also urgently needed to obtain data representative of Spanish and European markets.

## Figures and Tables

**Table 1:** Description of the ANIBES study sample (18–45 years).

		Total *n* = 641	%	Younger Women *n* = 251	%	Older Women *n* = 390	%
Education Level	Primary or less	122	19.0	43	17.1	79	20.3
	Secondary	329	51.3	130	51.8	199	51.0
	Tertiary or University	190	29.6	78	31.1	112	28.7
Habitat size	Rural	221	34.5	86	34.3	135	34.6
	Semi-urban	218	34.0	91	36.3	127	32.6
	Urban	202	31.5	74	29.5	128	32.8
Geographical distribution	Northeast	75	11.7	25	10.0	50	12.8
	Levante (East)	102	15.9	40	15.9	62	15.9
	South	140	21.8	56	22.3	84	21.5
	Central	50	7.8	25	10.0	25	6.4
	Northwest	55	8.6	21	8.4	34	8.7
	North central	67	10.5	33	13.1	34	8.7
	Canary Islands	35	5.5	14	5.6	21	5.4
	Madrid Metropolitan Area	77	12.0	28	11.2	49	12.6
	Barcelona Metropolitan Area	40	6.2	9	3.6	31	7.9

**Table 2:** Recommended dietary intakes (RDI) and adequate intake (AI) in women of childbearing age (18–45 years) for each micronutrient according to Spain (Moreiras et al. [1]), Europe (EFSA) [2] and USA (IOM) [3].

	Spain	Europe	USA
AI for choline (mg/d)	-	400	425
RDI for vitamin B_6_ (mg/d)	1.6	1.6	1.3
RDI for folic acid (µg/d)	400	330	400
RDI for vitamin B_12_ (µg/d)	2	4	2.4

**Table 3:** Choline intake and prevalence of adequacy (percentage of population above 80% AI) in women of childbearing age from the ANIBES study.

	Choline (mg/d)	% Above 80% AI EFSA	% Above 80% AI IOM
Total *n* = 641	303.9 (243.2–373.6)	39.5	35.1
Younger women *n* = 251	292.4 * (236.4–363.1)	35.1	31.1
Older women *n* = 390	311.8 (256.0–378.0)	42.3	37.7

AI: Adequate Intake. Values are median (interquartile range) per group. * *p* < 0.05 difference younger women vs. older women (Mann-Whitney’s U test).

**Table 4:** Betaine intake in women of childbearing age from the ANIBES study.

	Betaine (mg/d)
Total *n* = 641	122.6 (90.7–159.0)
Younger women *n* = 251	113.5 *** (86.5–145.1)
Older women *n* = 390	130.2 (96.4–168.7)

Values are median (interquartile range) per group. *** *p* < 0.001 difference younger women vs. older women (Mann-Whitney’s U test).

**Table 5:** Vitamin B_6_ intake and prevalence of adequacy (percentage of population above 80% RDI) in women of childbearing age from the ANIBES study.

	Vitamin B_6_ (mg/d)	% Above 80% RDI SPAIN	% Above 80% RDI EFSA	% Above 80% RDI IOM
Total *n* = 641	1.3 (1.0–1.7)	52.1 ^a^	59.3 ^b^	79.4 ^c^
Younger women *n* = 251	1.3 (1.0–1.6)	50.6 ^a^	57.0 ^a^	74.1 ^b^
Older women *n* = 390	1.3 (1.0–1.7)	53.1 ^a^	60.8 ^b^	75.4 ^c^

RDI: Recommended Dietary Intakes. Values are median (interquartile range) per group. Different superscript lowercase letters (a, b, and c) indicate statistical significance in each row between different RDI (*p* ≤ 0.05; Chi-Square test).

**Table 6:** Folates intake and prevalence of adequacy (percentage of population above 80% RDI) in women of childbearing age from the ANIBES study.

	Folates (µg/d)	% Above 80% RDI Spain	% Above 80% RDI EFSA	% Above 80% RDI IOM
Total *n* = 641	140.8 (105.5–186.5)	2.3 ^a^	21.4 ^b^	2.3 ^a^
Younger women *n* = 251	131.4 * (102.8–181.1)	1.6 ^a^	18.7 ^b^	1.6 ^a^
Older women *n* = 390	148.3 (108.7–191.0)	2.8 ^a^	23.1 ^b^	2.8 ^a^

RDI: Recommended Dietary Intakes. Values are median (interquartile range) per group. * *p* < 0.05 difference younger women vs. older women (Mann–Whitney’s U test). Different superscript lowercase letters (a, b) indicate statistical significance in each row between different RDI (*p* < 0.001; chi-square test).

**Table 7:** Vitamin B_12_ intake and prevalence of adequacy (percentage of population above 80% RDI) in women of childbearing age ANIBES study.

	Vitamin B_12_ (µg/d)	% Above 80% RDI Spain	% Above 80% RDI EFSA	% Above 80% RDI IOM
Total *n* = 641	3.8 (2.5–5.3)	91.0 ^a^	62.2 ^b^	86.1 ^c^
Younger women *n* = 251	3.7 (2.4–5.4)	90.4 ^a^	59.8 ^b^	84.9 ^a^
Older women *n* = 390	4.0 (2.6–5.2)	91.3 ^a^	63.8 ^b^	86.9 ^a^

RDI: Recommended Dietary Intakes. Values are median (interquartile range) per group. Different superscript lowercase letters (a, b, and c) indicate statistical significance in each row between different RDI (*p* < 0.01; chi-square test).

## Data Availability

The data presented in this study are available on request from the corresponding author.
